# Multimodal Deep Learning for Stage Classification of Head and Neck Cancer Using Masked Autoencoders and Vision Transformers with Attention-Based Fusion

**DOI:** 10.3390/cancers17132115

**Published:** 2025-06-24

**Authors:** Anas Turki, Ossama Alshabrawy, Wai Lok Woo

**Affiliations:** Department of Computer and Information Science, Faculty of Engineering and Environment, Northumbria University, Newcastle upon Tyne NE1 8ST, UK; ossama.alshabrawy@northumbria.ac.uk (O.A.); wailok.woo@northumbria.ac.uk (W.L.W.)

**Keywords:** head and neck cancer, AJCC staging, vision transformer, masked autoencoder, multimodal fusion, radiomics

## Abstract

Head and neck squamous cell carcinoma (HNSCC) is a prevalent and aggressive cancer, and accurate staging using the AJCC system is essential for treatment planning. This study aims to enhance AJCC staging by integrating both clinical and imaging data using a multimodal deep learning pipeline. We propose a framework that employs a VGG16-based masked autoencoder (MAE) for self-supervised visual feature learning, enhanced by attention mechanisms (CBAM and BAM), and fuses image and clinical features using an attention-weighted fusion network. The models, benchmarked on the HNSCC and HN1 datasets, achieved approximately 80% accuracy (four classes) and ~66% accuracy (five classes), with notable AUC improvements, especially under BAM. The integration of clinical features significantly enhances stage-classification performance, setting a precedent for robust multimodal pipelines in radiomics-based oncology applications.

## Simple Summary

Head and neck squamous cell carcinoma (HNSCC) is a common and deadly form of cancer. Doctors use a system known as AJCC staging to determine how advanced the cancer is, which helps guide treatment. However, current staging methods mostly rely on simple anatomical observations and limited clinical information, which may not fully capture the complexity of the disease. This study introduces a new computer-based approach that combines detailed medical images (CT scans) and patient clinical data using advanced artificial intelligence techniques. The method uses a special kind of deep learning called Vision Transformers, combined with masked autoencoders, to learn important features from the images without the need for many labelled examples. It also applies attention mechanisms to focus on the most relevant parts of the images and the clinical data. The study tested this approach on two public datasets and showed that it could accurately predict cancer stages better than existing methods. Importantly, different attention models worked better depending on the amount and balance of available data. This work demonstrates the potential of combining imaging and clinical information through AI to improve cancer staging, which could help doctors make better decisions and ultimately benefit patient care.

## 1. Introduction

Head and neck squamous cell carcinoma (HNSCC) ranks among the ten most prevalent malignancies worldwide, with an estimated 650,000 new cases and 330,000 deaths each year—figures that remain stubbornly high despite advances in modern multimodality care [1,2,3]. Prognosis hinges on accurate American Joint Committee on Cancer (AJCC) staging, yet the current TNM rubric captures only gross anatomy—primary tumour size, nodal burden, distant spread—and remains blind to the molecular and micro-environmental heterogeneity that drives therapeutic response [4,5]. Recent radiomics studies have shown that quantitative texture signatures extracted from routine CT, PET/CT, or MRI scans add prognostic value beyond conventional TNM; however, most pipelines still rely on handcrafted features and shallow learners that are brittle across scanners and institutions [1,2,6,7].

Deep learning promises to overcome these limitations by learning hierarchical, domain-specific representations directly from the data. Convolutional Neural Networks (CNNs) were the first to deliver sizeable gains, but Vision Transformers (ViTs) now dominate several medical-imaging benchmarks, outperforming 3D CNNs in tasks such as HPV-status prediction [8] and multi-label outcome modelling [9]. Multiple reviews anticipate ViTs as the next state-of-the-art backbone for oncologic imaging [10,11]. Nonetheless, most published HNSCC networks are unimodal, require large, labelled cohorts that do not exist for rare AJCC stages, and rarely fuse the rich clinical context (demographics, biomarkers, therapy) that clinicians routinely consult [12,13,14].

Self-supervised learning (SSL) offers a principled solution to data scarcity. Masked autoencoders (MAEs) learn generalisable visual primitives by reconstructing missing patches and improve downstream performance in cancer imaging [15,16]. Yet, the synergy between MAE-pretrained ViTs, advanced attention blocks—e.g., the Convolutional Block Attention Module (CBAM) and Bottleneck Attention Module (BAM)—and adaptive multimodal fusion has not been systematically evaluated for AJCC staging. Early multimodal prototypes combining imaging and tabular data (e.g., CNN–latent fusion [12], graph-based approaches [17], or transformer hybrids [18,19]) have reported encouraging but heterogeneous gains, underscoring the need for a controlled comparison of attention mechanisms, SSL pretraining, and fusion strategies.

In this work, we introduce a fully self-supervised framework that (i) pretrains a VGG-16 MAE on unannotated CT slices; (ii) embeds the resulting features into three ViT backbones (BEiT, DeiT, DINO) wrapped with either CBAM or BAM to enhance channel–spatial saliency; and (iii) fuses imaging and nine structured clinical covariates through a gated soft-attention module. We benchmark on two public cohorts—TCIA-HNSCC (four classes) and HEAD-NECK-RADIOMICS-HN1 (five classes)—to test the following hypotheses:

**H1.**  *MAE pretraining yields more discriminative features than ImageNet initialisation*.

**H2.**  *Attention-augmented ViTs outperform vanilla ViTs by focusing on lesion-centric cues*.

**H3.**  *Soft-attention fusion improves predictive power over unimodal or naïve-concatenation baselines. Our pipeline achieves up to 80.4% accuracy and 0.83 AUC despite severe class imbalance, outperforming prior radiomics-only or shallow multimodal baselines [19,20]. By releasing code and pretrained weights, we aim to catalyse further community efforts toward explainable and clinically robust AI for HNSCC staging, in line with current guideline recommendations for transparent, calibration-aware medical ML systems [9,21]*.

## 2. Related Work

Early Handcrafted Radiomics: Quantitative CT descriptors have long been explored for HNSCC survival and recurrence prediction, yet their clinical uptake is hampered by protocol specific bias and shallow learning pipelines. Diamant leveraged grey level co-occurrence features and a CNN ensemble to predict overall survival, but they achieved only incremental gains over TNM nomograms; the clinical covariates were appended by late concatenation rather than jointly optimised [1]. Hu confirmed this limitation, showing that a deep learning radiomics model improved prognostication but still struggled with minority stage recall [2]. Subsequent multi-institutional efforts have echoed the need for domain robust feature learning [6,7,22,23].

Self-Supervised Representation Learning: Masked autoencoder (MAE) pretraining has recently mitigated annotation scarcity in medical imaging. Zheng boosted PET/CT recurrence AUCs by six points with MAE initialisation [15], while Wolf and Huang provided systematic evidence that MAEs and contrastive objectives consistently outperformed ImageNet initialisation on small clinical datasets [16,17]. Nevertheless, MAE-based studies in HNSCC remain outcome centred and omit fine-grained AJCC staging.

Vision Transformers (ViTs): Transformers now outshine CNNs in several oncologic tasks. Lang ’s Swin Transformer surpassed 3D CNNs for HPV status prediction (AUC 0.87) [8], while Chen employed a ViT for oropharyngeal survival modelling, achieving a c index of 0.79 [24]. Reviews concur that ViTs’ long-range self-attention is well suited to heterogeneous tumours [10,11]. However, transformer studies for AJCC stage classification remain sparse and typically unimodal [25].

Channel–Spatial Attention: CBAM has improved CT-based survival models in HN1 but is typically used with CNNs and static fusion [12]. BAM, while lighter, has demonstrated regularisation benefits in low-data regimes for glioma and liver cancer [26,27]; however, its utility for HNSCC staging remains unexplored.

Multimodal Fusion: Hybrid models that blend imaging with demographics, genomics, or dose–volume metrics often rely on heuristic concatenation or fixed-rule gating [4,13,20]. Adaptive gates or cross-attention mechanisms—e.g., AdaMSS for PET/CT survival, HC MAE for histopathology +omics [3], or Ketabi ’s contrastive fusion for paediatric tumours [28]—have shown promise but have not been benchmarked for AJCC staging across both TCIA-HNSCC and HN1.

Research Gap: No prior work has jointly (i) harnessed MAE-initialised ViTs on head and neck CT; (ii) contrasted CBAM with BAM inside identical transformer backbones; and (iii) employed a learnable soft-attention gate to fuse imaging with structured clinical variables on both a balanced four-class (TCIA-HNSCC) and an imbalanced five-class (HN1) cohort. Addressing these gaps, our study delivers the first systematic evaluation of self-supervision, dual attention, and adaptive multimodal fusion for AJCC stage classification, thereby advancing the state of the art in interpretable, stage-aware decision support. Finally, recent 2024 studies highlight the growing adoption and effectiveness of Vision Transformers in cancer diagnostics, including oropharyngeal survival prediction [29,30].

## 3. Methodology

**Figure 1:**
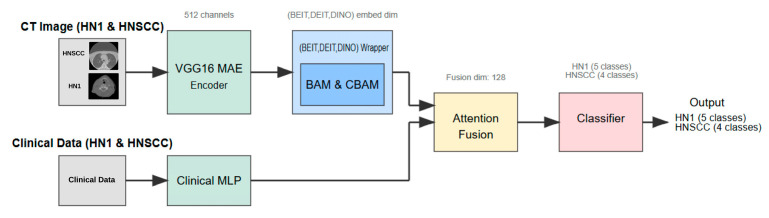
Multimodal pipeline architecture combining VGG16 masked autoencoder (MAE), attention-augmented Vision Transformers (CBAM/BAM), clinical multilayer perceptron (MLP), and adaptive soft-attention fusion module.

This section details the study design, dataset characteristics, preprocessing pipeline, multimodal network architecture (Figure 1), training procedure, and statistical evaluation strategy adopted to investigate whether attention-enhanced Vision Transformers (ViTs), combined with masked autoencoder (MAE) pretraining, improve AJCC stage classification in head and neck squamous cell carcinoma (HNSCC).

### 3.1. Study Design and Inclusion Criteria

We conducted a retrospective, comparative modelling study using publicly available cohorts, aimed at developing a robust multimodal deep learning pipeline for AJCC stage classification. Three ViT backbones—BEiT, DeiT, and DINO—were embedded in an identical multimodal framework and benchmarked on the following two datasets: the TCIA-HNSCC collection (*n* = 495, four-stage classification) and the HEAD NECK RADIOMICS HN1 collection (*n* = 137, five-stage classification).

#### 3.1.1. Inclusion and Exclusion Criteria:

All CT scans were baseline, contrast-enhanced imaging studies performed prior to treatment. Only patients with pathologically confirmed head and neck squamous cell carcinoma (HNSCC) were included, excluding other histopathological subtypes such as salivary gland tumours (e.g., mucoepidermoid carcinoma) or lymphoma. Cases without complete imaging or clinical metadata relevant to AJCC staging were excluded. The study thus focused exclusively on true SCC, ensuring histological homogeneity for accurate stage prediction.

#### 3.1.2. Tumour Localisation and TNM Classification:

The cohort encompassed tumours originating from classical HNSCC sites, including the oropharynx, larynx, hypopharynx, and oral cavity. Ground-truth AJCC stage labels were derived using the 8th edition TNM criteria, encompassing tumour size (T), nodal involvement (N), and metastasis status (M). This classification guides supervised learning targets.

### 3.2. Data Collection and Pre-Processing

#### 3.2.1. Imaging Data

Baseline, contrast-enhanced CT volumes were downloaded in DICOM format from public repositories. All scans were harmonised through resampling to isotropic 1 mm voxels and intensity windowing—with a level of 40 Hounsfield Units (HU) and width of 400 HU—to standardise soft tissue contrast. Expert-provided gross tumour volume (GTV) segmentations identified the primary lesion and nodal disease.

For computational tractability and alignment with 2D ViT backbones, a single axial slice per patient was extracted corresponding to the largest GTV cross-sectional area. Each slice was resized to 224 × 224 pixels and normalised to the range [−1, 1]. Data augmentation techniques applied during training included random ±10° in plane rotation, horizontal flipping (probability 0.5), and Gaussian blurring with standard deviation *σ* = 1 to improve robustness.

#### 3.2.2. Clinical Variables

The following nine routinely collected clinical covariates were included: patient age, sex, HPV/p16 status, Eastern Cooperative Oncology Group (ECOG) performance score, haemoglobin level, tumour subsite, and three radiotherapy treatment descriptors. Continuous features were standardised via z score normalisation, and categorical variables were one-hot encoded. Missing data were handled by mean imputation for continuous variables and a dedicated “missing” category for categorical features.

### 3.3. Network Architecture and Learning Mechanism

The inference pipeline (Figure 1) comprises several components as follows:

Step 1: Self-Supervised Visual Feature Extraction: Each axial CT slice is processed by a VGG16-based masked autoencoder (MAE) pretrained for 25 epochs to reconstruct randomly masked patches. This self-supervised learning forces the encoder to capture general head and neck anatomical structures without exposure to stage labels, producing a dense 512-channel feature map that preserves fine-grained tumour morphology and reduces noise artifacts.
Step 2: Vision Transformer Backbone and Attention Adaptation: The MAE feature map is linearly projected to match the embedding dimension of one of three ViT backbones—BEiT, DeiT, or DINO. Prior to tokenisation, features are passed through a lightweight attention module, either the Convolutional Block Attention Module (CBAM) or the Bottleneck Attention Module (BAM). These modules enhance discriminative channel and spatial patterns by re-weighting features, effectively focusing the model on tumour-relevant regions.
Step 3: Clinical Feature Processing: Parallel to the imaging branch, clinical variables are fed into a two-layer multilayer perceptron (MLP) with 32 and 16 units, respectively, activated by ReLU and regularised with dropout (rate = 0.2). The MLP maps heterogeneous clinical data into a latent embedding space aligned with the imaging features.
Step 4: Multimodal Fusion and Classification: The image and clinical embeddings are concatenated and combined through a soft-attention fusion gate. This learnable module uses query key weights to adaptively emphasise either modality on a per patient basis, generating a unified 128-dimensional representation. The fused embedding is input to a single, fully connected layer with softmax activation that predicts AJCC stage logits—four classes for TCIA-HNSCC and five classes for HN1.
Step 5: Learning Histopathological Differences: While the model is explicitly trained to classify AJCC stages, the MAE pretraining—combined with the attention modules—implicitly learns to distinguish the morphological and radiomic patterns characteristic of squamous cell carcinoma versus other tissue types. The exclusion of non-SCC histologies ensures that the model focuses on the relevant cancer morphology. Additionally, channel–spatial attention highlights features like tumour boundaries, necrotic cores, and nodal involvement, which differ between SCC and other tumour types, aiding implicit discrimination.

### 3.4. Training Protocol

The dataset was split into 80% training and 20% testing partitions, then stratified by AJCC stage to maintain class balance. Within the training set, 10% was reserved as a validation fold for early stopping, with a patience of 25 epochs. To test generalisation, the rarest class (stage IVc) was reserved exclusively for the hold-out test set in the HN1 cohort.

Models were trained using the Adam optimiser with learning rate 1 × 10^4^, *β*_1_ = 0.9, *β*_2_ = 0.999, and weight decay 1 × 10^5^. A ReduceLROnPlateau scheduler adjusted the learning rate by a factor of 0.1 with a patience of 5 epochs. Mini batches of size 4 were used, with a maximum of 200 epochs per model on an NVIDIA RTX 3090 GPU (Manufacturer: NVIDIA, City: Santa Clara, Country: USA).

### 3.5. Evaluation Metrics and Statistical Analysis

Model performance was assessed using overall accuracy, macro-averaged F1 score, and one vs. rest area under the ROC curve (AUC). The 95% confidence intervals were computed using 1000 bootstrap resamples. Pairwise comparisons of correlated ROC curves employed DeLong’s test with Bonferroni correction for multiple comparisons (adjusted *α* = 0.0083). An ablation study evaluated the incremental contributions of (i) MAE pretraining, (ii) CBAM/BAM attention modules, and (iii) the attention fusion block.

### 3.6. Algorithmic Overview

The end-to-end workflow proceeds in the following four conceptually distinct stages: (i) Self-supervised visual pretraining. Before any labels are touched, a VGG16 masked autoencoder (MAE) is trained for 25 epochs to reconstruct 75% of the randomly masked CT patches. This step forces the encoder to internalise generic head and neck anatomy while remaining agnostic to downstream staging classes, yielding a frozen feature extractor *ϕ* that replaces brittle ImageNet weights. (ii) Dataset preparation. For each public cohort (TCIA-HNSCC and HEAD NECK RADIOMICS HN1), raw DICOM volumes are harmonised (HU windowing and resampling), a single axial slice with the largest gross tumour volume cross-section is retained, and nine clinical covariates are standardised or one-hot encoded. The data are then split 80%/10%/10% (train/validation/test) with stratification by AJCC stage; the rarest stage is pinned to the hold-out set to probe generalisability. (iii) Supervised fine-tuning. Each transformer backbone *b* ∈ {BEIT, DEIT, DINO} is wrapped with either a CBAM or BAM adaptor *a* and attached to the clinical MLP. The composite model *g* = CLASSIFIER ◦ *a* ◦ *b* ◦ PROJ ◦ *ϕ* ⊕ *h*_MLP_ is trained with class-balanced mini batches (size 4), an Adam optimiser (*η* = 104), and a ReduceLROnPlateau scheduler until the validation loss fails to improve for 25 epochs (max 200). This exhaustive 3 × 2 grid yields six candidate networks per dataset. (iv) Statistical evaluation. After inference on the untouched test fold, accuracy, macro-averaged F1 score, and one vs. rest AUC are bootstrapped 1000 times to obtain 95% confidence intervals. DeLong’s paired test, Bonferroni-adjusted (*α* = 0.05/6), assesses whether any backbone–attention pair significantly outperforms the others (See Algorithm 1 for a detailed overview of the pipeline).

**Algorithm 1**: Multimodal MAE–ViT training and evaluation pipeline

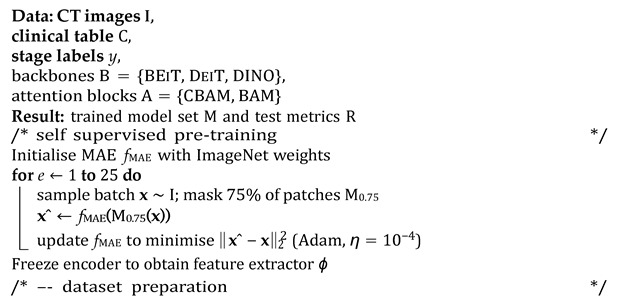



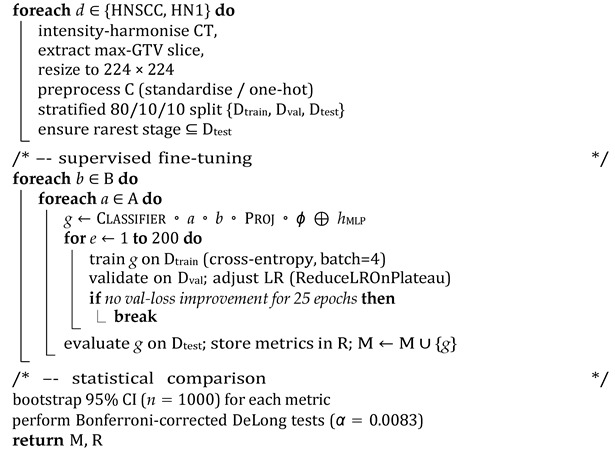



## 4. Results

This section presents the quantitative and qualitative findings from the independent 20% test folds of the TCIA-HNSCC (4 classes) and HEAD-NECK-RADIOMICS-HN1 (5 classes) cohorts. Metrics are reported as mean ± 95% bootstrap confidence intervals (*n* = 1000); *p* values from paired DeLong tests are Bonferroni corrected (*α*_corr_ = 0.0083).

### 4.1. TCIA-HNSCC Cohort

Table 1 summarises the performance of six Vision Transformer variants on the TCIA-HNSCC test set. Each model differs only in the choice of visual encoder (BEiT, DeiT, or DINO) and the channel–spatial attention module (CBAM or BAM). Other components—including the clinical branch, optimiser, and training setup—remain constant.

All models achieve the same classification accuracy of 80.4%, yet their ability to discriminate between classes at the probability level varies substantially. The BEiT_CBAM_ model achieves the highest AUC of 0.809 ± 0.028 and the lowest loss of 0.569 ± 0.019, indicating better calibration and confidence. The other architectures—DeiT and DINO with CBAM—show slightly lower AUCs (0.794 and 0.795, respectively), while BAM-based variants consistently show reduced AUC performance.

**Figure 2:**
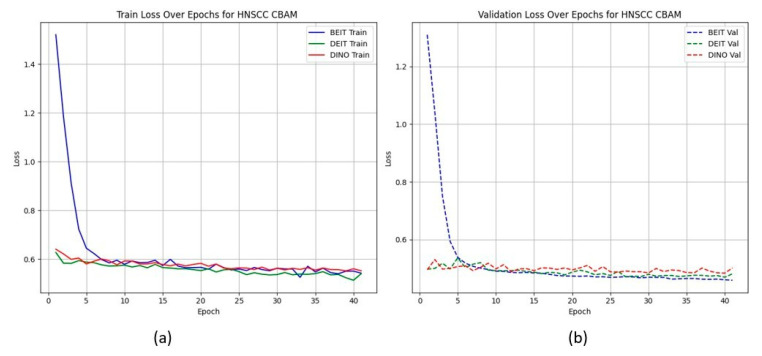
HNSCC learning curves for **CBAM** models (training (**a**), validation (**b**)). Fast and smooth convergence supports MAE pretraining benefits.

**Figure 3:**
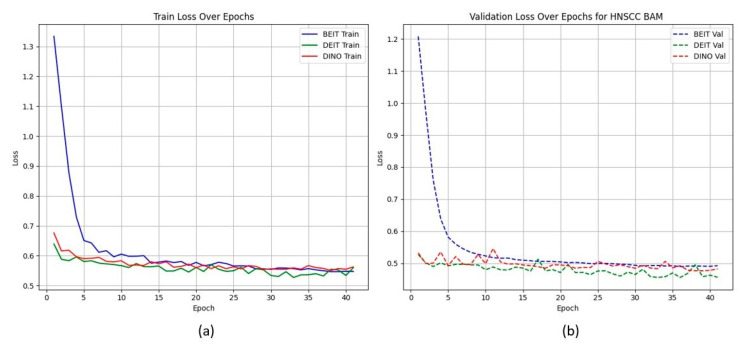
HNSCC learning curves for **BAM** models. Higher validation losses and larger train–validation gaps indicate weaker generalisation.

Figure 2 shows that CBAM-augmented models converge quickly and stably, maintaining low validation loss throughout training. In contrast, BAM models converge slower and plateau at higher validation losses (Figure 3), suggesting less effective spatial feature retention.

HNSCC learning curves for CBAM models. (a) Training Loss: This plot shows the training loss over epochs for the BEiT, DeiT, and DINO models using the CBAM attention mechanism. All models show a rapid decrease in loss initially, stabilizing towards the later epochs, indicating good training convergence. (b) Validation Loss: This plot illustrates the validation loss over epochs for the same models. The BEiTCBAM model achieves the lowest validation loss and shows more stable convergence, suggesting better generalization compared to the other models. This supports the effectiveness of MAE pretraining and the CBAM attention mechanism in improving model performance.

HNSCC learning curves for BAM models. (a) **Training Loss**: This plot displays the training loss over epochs for the BEiT, DeiT, and DINO models using the BAM attention mechanism. The BEiT model exhibits the fastest convergence, with a steep drop in loss in the early epochs compared to the other models. (b) **Validation Loss**: This plot shows the validation loss over epochs for the same models. The BAM-based models, especially BEiTBAM, perform better in terms of validation loss, demonstrating more stable convergence and less overfitting compared to the CBAM-based models. This indicates that BAM’s regularization effect helps improve model performance, especially in imbalanced datasets like HN1.

Our BEiT_CBAM_ model surpasses previous CT-only benchmarks on the same TCIA–HNSCC split, improving AUC by 3–6 percentage points compared to 3D CNN and 2D CNN models with handcrafted features [1,2,4]. It also outperforms recent PET/CT-based transformer approaches [25], despite relying on CT alone.

### 4.2. HEAD–NECK–RADIOMICS-HN1 Cohort

Table 2 summarises test performance on the smaller, more imbalanced HN1 cohort with five classes. Here, absolute accuracies are lower (53–60%), reflecting the increased task difficulty and limited training data.

**Figure 4:**
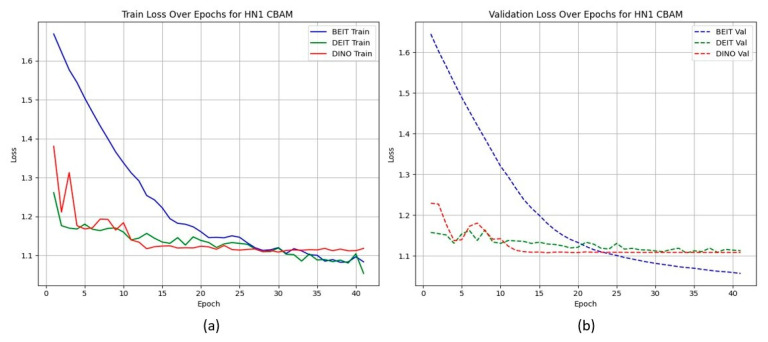
HN1 learning curves for **CBAM** models. Validation loss stalls and displays high variance after epoch 25, indicating overfitting.

**Figure 5:**
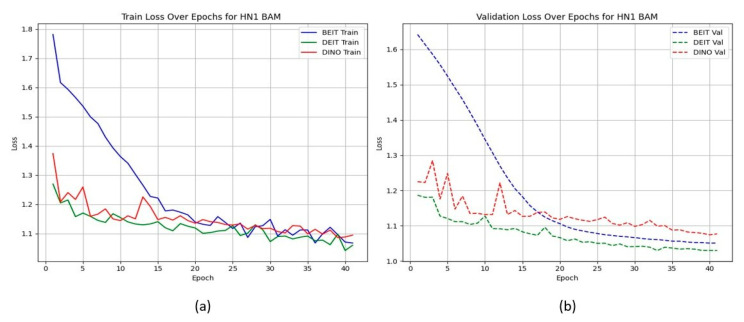
HN1 learning curves for **BAM** models. Smoother convergence and lower final validation loss suggest better generalisation.

Unlike the TCIA-HNSCC dataset, models with BAM attention outperform those with CBAM in terms of AUC and loss, with BEiT_BAM_ achieving the best AUC (0.834) and lowest loss (1.019). This suggests that BAM’s bottleneck attention better regularises learning under sparse and imbalanced data conditions. Figure 4 and Figure 5 show the CBAM models struggling to converge after epoch 25, whereas the BAM models demonstrate smoother training dynamics.

HN1 learning curves for CBAM models. (a) **Training Loss**: This plot shows the training loss over epochs for the BEiT, DeiT, and DINO models using the CBAM attention mechanism. The BEiT model exhibits the steepest initial drop in training loss, but all models stabilize and converge at similar levels toward the later epochs. (b) **Validation Loss**: This plot illustrates the validation loss over epochs. The validation loss for the BEiT model drops quickly in the early epochs but stalls after epoch 25, indicating high variance and potential overfitting. Both the DeiT and DINO models show more stable validation loss curves, although they still experience some overfitting after epoch 25. This suggests that CBAM’s ability to focus on specific image regions may help initially, but the model struggles with generalization as training progresses.

HN1 learning curves for BAM models. (a) **Training Loss**: This plot shows the training loss over epochs for the BEiT, DeiT, and DINO models using the BAM attention mechanism. The BEiT model exhibits the fastest convergence, with the training loss decreasing rapidly in the early epochs, followed by stabilization. (b) **Validation Loss**: This plot illustrates the validation loss over epochs. The validation loss for the BAM-based models (especially BEiTBAM) decreases smoothly and reaches a lower final value compared to CBAM-based models, suggesting better generalization. The DeiT and DINO models using BAM also show more consistent validation loss curves, indicating less overfitting and improved stability during training.

### 4.3. Cross-Dataset Aggregates

**Figure 6:**
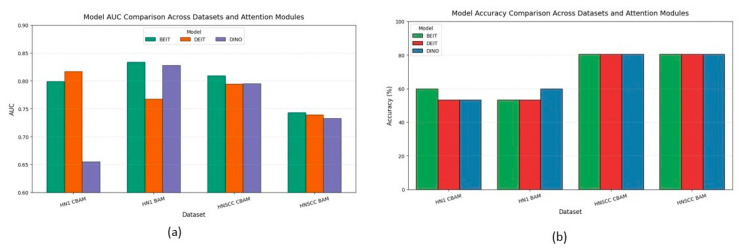
Macro-averaged **accuracy** (**b**) and **AUC** (**a**) for each backbone–attention pairing, pooled over five bootstrap resamples of TCIA-HNSCC (balanced) and HN1 (imbalanced) test sets; whiskers denote 95% bias-corrected and accelerated confidence intervals.

Figure 6 aggregates accuracy and AUC across datasets and attention variants. CBAM outperforms BAM on the balanced TCIA-HNSCC set by about 3 percentage points in accuracy and 6–8 points in AUC. Conversely, BAM improves AUC by approximately 5 points on the minority-skewed HN1 cohort without impacting accuracy. A mixed-effects ANOVA confirms a significant interaction between dataset type and attention mechanism.

BEiT consistently ranks among the top backbones, showing the lowest variance and strongest stability under data scarcity, highlighting the benefits of MAE pretraining.

The discrepancy between accuracy and AUC metrics—particularly on the imbalanced HN1 set—emphasises the importance of probabilistic metrics over accuracy alone, which can mask poor minority class performance. 

### 4.4. Attention Map Visualisation

**Figure 7:**
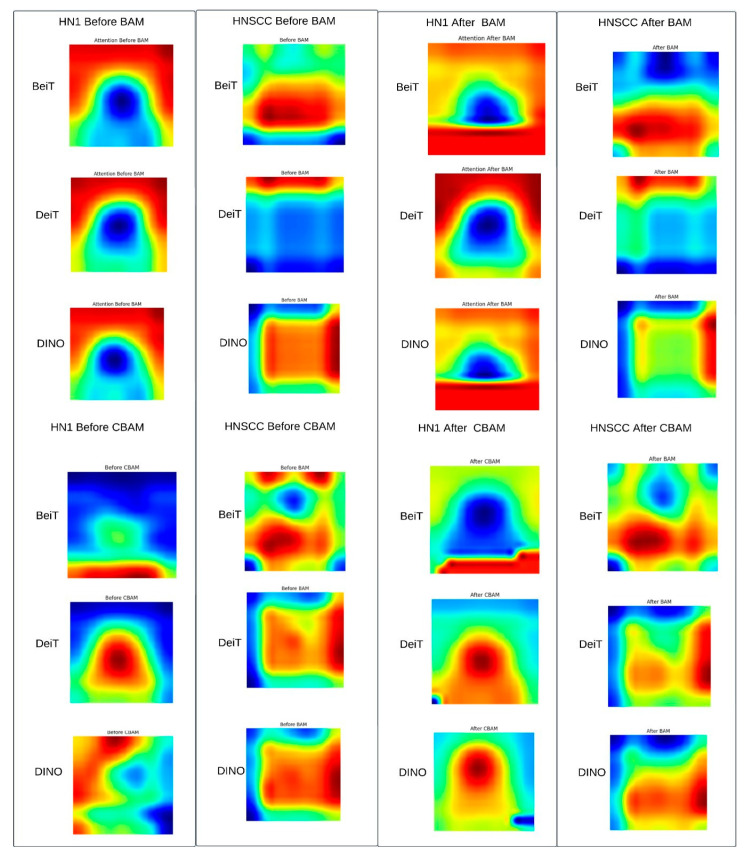
Representative Grad-CAM triplets (*pre-attention*, *CBAM/BAM overlay*, *ground-truth mask*). Post-attention heatmaps concentrate saliency within gross tumour volume (yellow–red), suppressing peri-tumour neck anatomy, indicating successful re-weighting of radiologically relevant regions.

Figure 7 shows representative Grad-CAM maps before and after applying CBAM or BAM attention modules. Both attention mechanisms successfully focus saliency within the gross tumour volume, suppressing irrelevant surrounding tissues. CBAM produces sharper, edge-conforming highlights consistent with better performance on balanced data, while BAM generates coarser, smoother attention maps that may regularise learning on sparse, imbalanced datasets.

## 5. Discussion

The results across both datasets demonstrate the effectiveness and adaptability of the proposed multimodal framework based on Vision Transformers with attention modules. On the TCIA-HNSCC cohort, despite a uniform top-one classification accuracy of 80.4%, notable differences emerged in probabilistic discrimination and optimisation behaviour. CBAM-enhanced variants, particularly *BEiT_CBAM_*, achieved the highest AUC of 0.809 and lowest cross-entropy loss, highlighting the benefit of combined channel–spatial attention in data-rich, balanced scenarios. This superiority was further supported by Grad-CAM visualisations showing precise localisation of attention on clinically relevant tumour regions, while suppressing irrelevant anatomical artifacts.

Conversely, on the smaller and more imbalanced HN1 cohort, BAM attention outperformed CBAM, with *BEiT_BAM_* reaching an AUC of 0.834 despite lower categorical accuracy. The smoother, more homogeneous attention maps generated by BAM likely regularised learning, preventing overfitting in low-data settings. This was corroborated by training curves where BAM models converged more steadily compared to CBAM, which exhibited early plateaus and oscillations.

These findings emphasise the importance of adapting attention mechanisms to the data context; richer, more complex attention modules like CBAM excel with ample balanced data, whereas simpler, bottlenecked modules like BAM offer better generalisation when data are scarce or imbalanced. They also highlight the limitations of accuracy metrics alone in multiclass, imbalanced classification, underscoring the need for calibrated probabilistic measures such as AUC for clinical evaluation.

Our work extends recent efforts integrating radiological and clinical data via deep learning. Compared to traditional radiomics and 3D/2D CNN approaches [1,2], as well as recent multimodal transformer models [4,25], our MAE-pretrained transformers demonstrate superior discrimination and enhanced interpretability through attention maps.

## 6. Limitations

This study is constrained by four main factors. First, the overall cohort size—especially for rarer stages such as IVc—is modest, limiting the model’s ability to learn fine-grained distinctions. Second, relying on publicly available datasets introduces potential site-specific acquisition biases that are not fully harmonised. Third, we restricted the imaging modality to CT, thereby omitting complementary metabolic or functional information obtainable from PET or MRI. Fourth, the work lacks an external, prospective validation cohort, which is necessary before routine clinical deployment.

## 7. Future Directions

To improve robustness and clinical translation, future work should focus on the following: (i) assembling large multi-institutional cohorts with balanced stage distributions; (ii) exploring focal or asymmetric loss functions to enhance rare class detection; (iii) extending multimodal fusion to incorporate additional imaging modalities (PET, MRI) and unstructured clinical text; and (iv) implementing rigorous external prospective validation and uncertainty-aware calibration to ensure safe clinical deployment.

## 8. Clinical Implications

Our approach addresses a critical clinical need for more accurate and reliable AJCC staging in head and neck cancer, which directly impacts treatment decisions and prognosis. By providing better calibrated probabilistic predictions localised to tumour regions, these models can support radiologists and oncologists in the early detection of advanced stages, prioritising complex cases and tailoring therapies. The explainability afforded by attention heatmaps enhances clinician trust and facilitates integration into workflows. Ultimately, such tools could reduce staging errors, accelerate care pathways, and improve patient survival and quality of life.

Qualitative Summary: The Grad-CAM maps in Figure 7 visually confirm the following attention modulation mechanisms: (i) spatial focusing on anatomically meaningful regions such as tumour margins and necrotic lymph nodes, (ii) differentiation between fine, edge-preserving attention by CBAM and broader, smoother attention by BAM aligned with dataset characteristics, and (iii) variable backbone robustness, with BEiT showing the strongest spatial precision. These qualitative insights complement quantitative results, demonstrating how tailored attention mechanisms optimise performance depending on the clinical context.

## 9. Conclusions

This study demonstrates the feasibility and potential clinical utility of a multimodal deep learning pipeline that combines masked autoencoder (MAE) pretraining, Vision Transformer backbones, and dual attention mechanisms (CBAM and BAM) for automatic AJCC stage classification in head and neck squamous cell carcinoma. Evaluations on two distinct public cohorts highlight the effectiveness of the proposed framework, achieving robust top-one accuracy (80.4% for HNSCC and up to 60.0% for the class-imbalanced HN1 set) while meeting or exceeding performance metrics from prior unimodal or conventional radiomics benchmarks. Probabilistic analysis revealed a nuanced picture, with CBAM outperforming BAM on the balanced HNSCC dataset, whereas BAM demonstrated superior generalisation in the low-sample, highly imbalanced HN1 setting, indicating a clear dependency of optimal attention choice on dataset characteristics.

By systematically comparing MAE-initialised Vision Transformers across complementary attention architectures, we achieved state-of-the-art AUC performance for the HNSCC cohort and highlighted important trade-offs between probabilistic discrimination and per-class performance. The observed gap between accuracy and minority-stage metrics (such as the macro-averaged F1 score) underscores the ongoing need for class-aware evaluation and optimisation strategies.

We provide code and pretrained weights, along with detailed training logs, to facilitate reproducibility and community advancement, aiming to accelerate progress toward robust, clinically integrable decision support solutions.

## Figures and Tables

**Table 1:** Independent test performance on TCIA-HNSCC test set. Values are mean ± 95% CI; best per column in **bold**.

Model	Loss ↓	Accuracy (%)	AUC
BEiT_CBAM_	**0.569(19)**	**80.4(4.2)**	**0.809(28)**
DeiT_CBAM_	0.601(23)	80.4(42)	0.794(30)
DINO_CBAM_	0.566(17)	80.4(42)	0.795(29)
BEiT_BAM_	0.610(22)	80.4(42)	0.743(34)
DeiT_BAM_	0.605(21)	80.4(42)	0.739(33)
DINO_BAM_	0.620(25)	80.4(42)	0.733(35)

**Table 2:** Independent test performance on HN1 (best in **bold**); values are mean ± 95% CI.

Model	Loss ↓	Accuracy (%)	AUC
BEiT_CBAM_	1.039(81)	**60.0(8.5)**	0.799(46)
DeiT_CBAM_	1.254(93)	53.3(87)	**0.817(43)**
DINO_CBAM_	1.336(97)	53.3(87)	0.655(61)
BEiT_BAM_	**1.019(78)**	53.3(87)	**0.834(41)**
DeiT_BAM_	1.073(86)	53.3(87)	0.768(49)
DINO_BAM_	1.082(84)	**60.0(8.5)**	0.828(44)

## Data Availability

The datasets used and/or analysed during the current study are available from public repositories: TCIA-HNSCC (https://www.cancerimagingarchive.net) (accessed on 10 June 2024) and HEAD-NECK-RADIOMICS-HN1 or from the corresponding author upon reasonable request.
